# Survey of Borreliae in ticks, canines, and white-tailed deer from Arkansas, U.S.A.

**DOI:** 10.1186/1756-3305-5-139

**Published:** 2012-07-10

**Authors:** Rebecca T Trout Fryxell, C Dayton Steelman, Allen L Szalanski, Ken L Kvamme, Peggy M Billingsley, Philip C Williamson

**Affiliations:** 1 Department of Entomology and Plant Pathology, University of Tennessee, Knoxville, TN, USA; 2 Department of Entomology, University of Arkansas, Fayetteville, AR, USA; 3 Department of Anthropology, University of Arkansas, Fayetteville, AR, USA; 4 Department of Forensic and Investigative Genetics, University of North Texas Health Science Center, Ft. Worth, TX, USA

**Keywords:** *Borrelia*, Ticks, Vector borne, Surveillance, Deer

## Abstract

**Background:**

In the Eastern and Upper Midwestern regions of North America, *Ixodes scapularis* (L.) is the most abundant tick species encountered by humans and the primary vector of *B. burgdorferi,* whereas in the southeastern region *Amblyomma americanum* (Say) is the most abundant tick species encountered by humans but cannot transmit *B. burgdorferi.* Surveys of Borreliae in ticks have been conducted in the southeastern United States and often these surveys identify *B. lonestari* as the primary *Borrelia* species, surveys have not included Arkansas ticks, canines, or white-tailed deer and *B. lonestari* is not considered pathogenic. The objective of this study was to identify *Borrelia* species within Arkansas by screening ticks (n = 2123), canines (n = 173), and white-tailed deer (n = 228) to determine the identity and locations of Borreliae endemic to Arkansas using PCR amplification of the flagellin (*flaB)* gene.

**Methods:**

Field collected ticks from canines and from hunter-killed white-tailed were identified to species and life stage. After which, ticks and their hosts were screened for the presence of *Borrelia* using PCR to amplify the *flaB* gene. A subset of the positive samples was confirmed with bidirectional sequencing.

**Results:**

In total 53 (21.2%) white-tailed deer, ten (6%) canines, and 583 (27.5%) Ixodid ticks (252 *Ixodes scapularis*, 161 *A. americanum*, 88 *Rhipicephalus sanguineus*, 50 *Amblyomma maculatum,* 19 *Dermacentor variabilis,* and 13 unidentified *Amblyomma* species) produced a *Borrelia flaB* amplicon. Of the positive ticks, 324 (22.7%) were collected from canines (151 *A. americanum,* 78 *R. sanguineus*, 43 *I. scapularis,* 26 *A. maculatum,* 18 *D. variabilis*, and 8 *Amblyomma* species) and 259 (37.2%) were collected from white-tailed deer (209 *I. scapularis,* 24 *A. maculatum,* 10 *A. americanum,* 10 *R. sanguineus*, 1 *D. variabilis*, and 5 *Amblyomma* species). None of the larvae were PCR positive. A majority of the *flaB* amplicons were homologous with *B. lonestari* sequences: 281 of the 296 sequenced ticks, 3 canines, and 27 deer. Only 22 deer, 7 canines, and 15 tick *flaB* amplicons (12 *I. scapularis*, 2 *A. maculatum*, and 1 *Amblyomma* species) were homologous with *B. burgdorferi* sequences.

**Conclusions:**

Data from this study identified multiple Borreliae genotypes in Arkansas ticks, canines and deer including *B. burgdorferi* and *B. lonestari;* however, *B. lonestari* was significantly more prevalent in the tick population than *B. burgdorferi*. Results from this study suggest that the majority of tick-borne diseases in Arkansas are not *B. burgdorferi.*

## Background

In the Eastern and Upper Midwestern regions of North America, *Ixodes scapularis* (L.) is the most abundant tick species encountered by humans and the primary vector of *Borrelia burgdorferi* (causative agent of Lyme disease)*,* whereas in the southeastern region *Amblyomma americanum* (Say) is the most abundant tick species encountered by humans but it cannot transmit *B. burgdorferi*[1,2]*.* Since its first description in the 1970s, Lyme disease is the most frequently reported vector-borne disease in the northern hemisphere [3]. However, in some areas of the world Lyme borreliosis may be caused by *Borrelia* genotypes other than *B. burgdorferi* and includes a range of symptoms and pathologies [3,4]. In the southern United States *B. lonestari* is associated with Southern Tick Associated Rash Illness (STARI) or Masters disease [5,6]. This bacteria is common throughout the southeast and has been identified primarily in *A. americanum;* however, the etiology of *B. lonestari* remains undetermined [7-15] and recent reports indicate *B. lonestari* may not be pathogenic [16].

Health professionals often question cases of Lyme disease from the southeastern United States because symptoms may be confused with other tick-borne illnesses and not all patients produce the erythema migrans or bull’s eye rash used for diagnosis [15,17]. Additionally, these cases are rarely fatal, but can cause cardiac, neurological and joint problems [17]. However, the potential vectors (*I. scapularis*), pathogens (*B. burgdorferi*) and hosts (*Peromyscus* species) are all present in the south [17]. In Georgia, *B. burgdorferi* was isolated and characterized from field collected *I. scapularis* and cotton mice (*Peromyscus gossypinus*); and these field isolates were transmitted via *I. scapularis* to hamsters (Cricetidae) and mice (Muridae) [1]. Field collected *I. scapularis* from Alabama could acquire, maintain, and transmit *B. burgdorferi*[18] and field collected ticks and rodents from the southern U.S. were positive for *B. burgdorferi*[1,19-22]. Previously in Arkansas, ticks and reservoir species were screened for *Borrelia* in nine northeast Arkansas counties using indirect fluorescence antibody (IFA), both *I. scapularis* and *A. americanum* were identified as potential vectors, and deer mice (*Peromyscus* spp.) and marsh rice rats (*Oryzomys palustris* Harlan) as potential reservoir hosts [23]. Although the environment is suitable for *Borrelia* transmission [17], many researchers and physicians do not believe the southern U.S. is at risk for *Borrelia* related diseases [2,4,15]. The exact cause for the reduced incidence for Lyme disease in the southern United States is unknown, but hypotheses include the abundance of other tick species in the area, the habitat, host dynamics, and tick genetics [2,24].

Previous research indicates that *B. burgdorferi* IFA positive canines geographically associated with *I. scapularis* infested deer, increase the likelihood of *B. burgdorferi* transmission to humans because the nymphs that feed on the deer may also feed on canines and their owners [25,26]. Recent Arkansas tick studies identified five tick species infesting canines and white-tailed deer with *A. americanum* infesting canines and *I. scapularis* primarily infesting white-tailed deer [27]. A population genetic study of *I. scapularis* based on the 16 S mtDNA identified both the American and Southern lineages (formerly known as *I. scapularis* and *I. dammini*) on canines and white-tailed deer [28], which is a concern because the American *I. scapularis* lineage is more likely than the Southern *I. scapularis* lineage to transmit Lyme disease [29]. The objective of this study was to identify *Borrelia* species within Arkansas ticks, canines, and white-tailed deer to determine the identity and locations of Borreliae endemic to Arkansas using PCR amplification of the flagellin (*flaB)* gene. The CDC defines an endemic county as “endemic for Lyme disease if there are at least two confirmed human cases that were acquired in that county or there are established populations of *I. scapularis* infected with *B. burgdorferi”*[30].

## Methods

### Tick, canine, and white-tailed deer collections

This project used the collections reported in Trout and Steelman [27]. Briefly, ticks were collected from canines (March 2006 to November 2007) and white-tailed deer (Oct. 2007 – Jan. 2008) and stored in 80% ethanol until visual identification of species and life stage [31-33]. Additionally, a small volume of canine or white-tailed deer blood (0.5-1 cc) was obtained from each host and stored on a FTA card (FTA ® Indicating Micro Card, Whatman International Ltd, Maidstone, England) in an envelope labeled with collection information. FTA cards were stored at room temperature until DNA extraction. All FTA cards, all ticks from canines, and at least one specimen of each tick species from each white-tailed deer were subjected to further analyses.

### Tick DNA extraction and detection for *Borrelia* species

To minimize DNA contamination, DNA extractions and PCR were conducted in different laboratories and reagents and equipment were dedicated to each procedure. Tick identification and extractions were conducted in the Veterinary Entomology Laboratory (VELUA) and PCR reactions were conducted in the Insect Genetics Laboratory (IGLUA) at the University of Arkansas. During each PCR, at least one blank reagent to detect contamination (negative control) and an appropriate positive control ensured PCR reagents and conditions were used. PCR and reaction product analyses were performed on the ticks according to the protocols of Trout *et al.*[28]. After tick identification to species, each specimen was dried on a paper towel to allow the residual ethanol to evaporate and was then cut longitudinally and half of the tick was subjected to the Qiagen Dneasy Insect Protocol (Qiagen Inc., Valencia, CA). Extracted DNA from half of the tick was stored at −20°C until further analyses. The remaining tick half was stored at −20°C for additional analysis or morphological confirmation as required. To evaluate the tick DNA, each sample was first assessed by PCR with Ixodidae specific mitochondrial primers (16 S + 2/16 S-1) using previously described cycling parameters [34]. If the mitochondrial marker amplified, then the tick was subjected to a genus specific *flaB* PCR to identify the presence of *Borrelia* DNA [6,35].

### Host DNA extraction and detection for *Borrelia* species

Each canine and white-tailed deer FTA card was screened in duplicate to determine the presence of *Borrelia* DNA*.* The FTA card was cut into halves, where half of the card remained at VELUA for an initial screening and the other half was sent to the University of North Texas Health Science Center in Ft. Worth (UNTHSC) for verification screening. Each FTA card half was removed from its envelope and a 1.2 mm disc was punched from the card using a sterilized Harris Micro Punch and the paper disc was washed for DNA extraction according to the Whatman protocol (Harris MicroPunch®, Whatman International Ltd, Maidstone, England). After the punch had dried at room temperature, it was subjected to PCR analyses. To ensure DNA detection from FTA punches were not inhibited in any manner, FTA punches were assessed for PCR amplification of host cytochrome b genes [36]. As with the ticks, canines and white-tailed deer specimens were screened for *Borrelia* species by PCR in a genus specific manner [6,35]*.*

### Statistical analyses

Summary statistics and relative abundance of each tick species were calculated to determine overall trends within the population using JMP (α = 0.05) [37]. Fisher’s exact tests and two-tailed T-tests were performed in Excel 2007 to determine if the species of tick or tick host had any effect on the probability that the tick would be PCR positive for *Borrelia*[38]. Since spatial and temporal sampling of ticks and hosts were different (e.g., ticks from white-tailed deer were collected only in the fall) and this sampling structure may affect findings, comparisons across and among cohorts were not conducted.

### Sequence identification of *Borrelia* species

PCR products, from *flaB* positive samples, were sent to UNTHSC for DNA sequence analysis. The PCR reaction products were hydrolyzed with ExoSAP-IT (USB Corporation, Cleveland, OH) and sequence determination was performed using a BigDYE Terminator v.3.1 Cycle Sequencing kit (Applied Biosystems, Inc., Foster City, CA) followed by capillary electrophoresis on a ABI PRISM 310 Genetic Analyzer (Applied Biosystems Inc., Foster City, CA) using the method of Williamson *et al*. [39]. Sequences were edited, aligned, and analyzed with Sequencher 4.7 (Gene Codes, Corporation, Ann Arbor MI) and compared with sequences in GenBank (National Center for Biotechnology Information, Bethesda, MD).

### Spatial identification of *Borrelia* species

Canine and deer collection data along with their corresponding tick collections and *Borrelia* DNA presence/absence data were georeferenced into ArcMap 9.0 (ESRI Redlands, CA USA) and projected to the NAD 1983 UTM Zone 15 N of the GCS North American 1983 Geographic coordinate system. Boolean operations and symbology were used to identify locations with PCR positive ticks and hosts. The resulting Boolean map was overlaid on an existing county map of Arkansas [40].

## Results

### Identification of *Borrelia* DNA in the tick population

**Figure 1:**
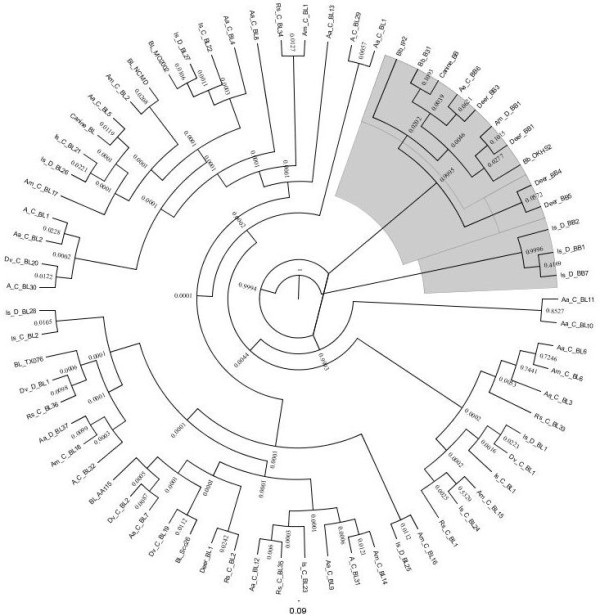
**Polar tree layout of the phylogenetic relationship of the*Borrelia*flagellin B (*flaB*) gene fragment (330 bp) amplified from ticks collected from Arkansas canines and white-tailed deer, and host blood samples**^**a**^**.** Those specimens aligning with *B. burgdorferi* are highlighted in gray^b^. ^a^ The different tick species are abbreviated *A. americanum* (Aa), *A. maculatum* (Am), *D. variabilis* (Dv), *I. scapularis* (Is), and *R. sangineus* (Rs). Ticks from canines (c), ticks from deer (d), and blood samples (Canine or Deer) are also represented. ^b^The different *Borrelia* species are abbreviated as BL for *B. lonestari* and BB for *B. burgdorferi*

**Figure 2:**
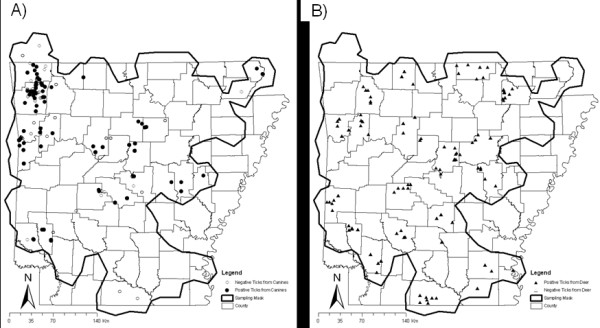
**Locations of*Borrelia flaB*amplicons isolated from ixodid ticks collected from Arkansas canines (A) and white-tailed deer (B) during 2007**

A total of 2123 ticks were included in this study and represented five tick species; *I. scapularis* (33%), *A. americanum* (31%), *R. sanguineus* (16 %), *D. variabilis* (9%), and *A. maculatum* (8%). An additional 3% were *Amblyomma* ticks that could not be identified to species because the tick specimens were damaged. The tick population was comprised of each life stage, but most were adults (83%) and more were collected from domesticated canines (67%) than from hunter-killed white-tailed deer (33%). Overall, 583 of the 2123 (27.5%) ticks tested by PCR produced an amplicon for the *Borrelia flaB* gene (Table 1). The prevalence of *flaB* amplicons for each tick species were 36.4 % (252/692) *I. scapularis*, 27.9% (50/179) *A. maculatum,* 25.8% (88/341) *R. sanguineus*, 24.5% (161/657) *A. americanum,* and 10.3% (19/184) *D. variabilis*. Fifty percent of the 583 *flaB* amplicons from PCR positive ticks were sequenced, representing approximately one fourth of the specimens from each tick species (Table 2). DNA sequences produced from the tick *flaB* amplicons represented multiple genotypes of *Borrelia*. These sequences align with *B. burgdorferi* strain B31 (AB035617), OK-strain HS-2 (FJ871032), *Borrelia lonestari* Clone Scc26 (DQ100451), isolate MO2002-V1 (AY850063), isolate TX076 (EF689742), strain NC/MD (AF273670), and clone AA115 (AY654945) (Figure 1). The majority (136/296) of *flaB* sequences matched *B. lonestari* strain NC/MD (AF273670) which was amplified from all five tick species. Locations of all *Borrelia* isolated from ixodid ticks collected from Arkansas canines and white-tailed deer during 2007 were mapped because *B. burgdorferi* ticks were few (Figure 2).

**Table 1:** **Almost a third of each Ixodid ticks**^**a**^**species collected from Arkansas canines and white-tailed deer generated amplicons by PCR for the*Borrelia flaB*gene indicating Borreliae are endemic to Arkansas**

**Tick species**	**No. PCR positive ticks / No. Screened for*flaB*(%)**					
	**Larvae**	**Nymphs**	**Males**	**Females**	**Adults**^a^	**Total**
Ticks Collected from Canines						
*Amblyomma* spp.	0 / 18 (0 %)	4 / 15 (26.7 %)	2 / 11 (18.2 %)	0 / 7 (0 %)	2 / 11 (18.2 %)	8 / 62 (12.9 %)
*Amblyomma americanum*	0 / 0 (0 %)	37 / 162 (22.8 %)	58 / 237 (24.5 %)	56 / 233 (24 %)	0 / 2(0 %)	151 / 634 (23.8 %)
*Amblyomma maculatum*	0 / 2 (0 %)	2 / 9 (22.2 %)	22 / 95 (23.2 %)	2 / 9 (22.2 %)	0 / 0 (0 %)	26 / 115 (22.6 %)
*Dermacentor variabilis*	0 / 1 (0 %)	0 / 41 (0 %)	7 / 52 (13.5 %)	11 / 84 (13.1 %)	0 / 0 (0 %)	18 / 178 (10.1 %)
*Ixodes scapularis*	0 / 0 (0 %)	3 / 7 (42.9 %)	21 / 42 (50 %)	18 / 69 (26.1 %)	1 / 3 (33.3 %)	43 / 121 (35.5 %)
*Rhipicephalus sanguineus*	0 / 0 (0 %)	28 / 97 (28.9 %)	19 / 94 (20.2 %)	22 / 105 (21 %)	9 / 21 (42.9 %)	78 / 317 (24.6 %)
Total	0 / 21 (0 %)	74 / 331 (22.4 %)	129 / 531 (24.3 %)	109 / 507 (21.5 %)	12 / 37 (32.4 %)	324 / 1427 (22.7 %)
Ticks Collected from White-tailed Deer						
*Amblyomma* spp.	0 / 0 (0 %)	4 / 7 (57.1 %)	0 / 0 (0 %)	1 / 1 (100 %)	0 / 0 (0 %)	5 / 8 (62.5 %)
*Amblyomma americanum*	0 / 0 (0 %)	0 / 2 (0 %)	2 / 7 (28.6 %)	8 / 14 (57.1 %)	0 / 0 (0 %)	10 / 23 (43.5 %)
*Amblyomma maculatum*	0 / 0 (0 %)	0 / 1 (0 %)	9 / 35 (25.7 %)	15 / 28 (53.6 %)	0 / 0 (0 %)	24 / 64 (37.5 %)
*Dermacentor variabilis*	0 / 0 (0 %)	0 / 2 (0 %)	0 / 2 (0 %)	1 / 2 (50 %)	0 / 0 (0 %)	1 / 6 (16.7 %)
*Ixodes scapularis*	0 / 1 (0 %)	4 / 6 (66.7 %)	86 / 231 (37.2 %)	119 / 333 (35.7 %)	0 / 0 (0 %)	209 / 571 (36.6 %)
*Rhipicephalus sanguineus*	0 / 0 (0 %)	0 / 0 (0 %)	5 / 10 (50 %)	5 / 14 (35.7 %)	0 / 0 (0 %)	10 / 24 (41.7 %)
Total	0 / 1 (0 %)	8 / 18 (44.4 %)	102 / 285 (35.8 %)	149 / 392 (38 %)	0 / 0 (0 %)	259 / 696 (37.2 %)
Total Ticks Collected from Canines and White-tailed Deer						
*Amblyomma* spp.	0 / 18 (0 %)	8 / 22 (36.4 %)	2 / 11 (18.2 %)	1 / 8 (12.5 %)	2 / 11 (18.2 %)	13 / 70 (18.6 %)
*Amblyomma americanum*	0 / 0 (0 %)	37 / 164 (22.6 %)	60 / 244 (24.6 %)	64 / 247 (25.9 %)	0 / 2 (0 %)	161 / 657 (24.5 %)
*Amblyomma maculatum*	0 / 2 (0 %)	2 / 10 (20 %)	31 / 130 (23.8 %)	17 / 37 (45.9 %)	0 / 0 (0 %)	50 / 179 (27.9 %)
*Dermacentor variabilis*	0 / 1 (0 %)	0 / 43 (0 %)	7 / 54 (13 %)	12 / 86 (14 %)	0 / 0 (0 %)	19 / 184 (10.3 %)
*Ixodes scapularis*	0 / 1 (0 %)	7 / 13 (53.8 %)	107 / 273 (39.2 %)	137 / 402 (34.1 %)	1 / 3 (33.3 %)	252 / 692 (36.4 %)
*Rhipicephalus sanguineus*	0 / 0 (0 %)	28 / 97 (28.9 %)	24 / 104 (23.1 %)	27 / 119 (22.7 %)	9 / 21 (42.9 %)	88 / 341 (25.8 %)
Total	0 / 22 (0 %)	82 / 349 (23.5 %)	231 / 816 (28.3 %)	258 / 899 (28.7 %)	12 / 37 (32.4 %)	583 / 2123 (27.5 %)

^**a**^ Damaged adult specimens could not be properly sexed.

**Table 2:** **Multiple Borreliae genotypes**^**a**^**were identified in Arkansas ticks**^**b**^**, canines and white-tailed deer including*B. burgdorferi*and*B. lonestari;*however,*B. lonestari*was significantly more prevalent than*B. burgdorferi*in the tick population**

**Host pecies**	**Seq. / PCR Pos. / screened**	**No. hosts*flaB*positive with amplicons homologous to*B. burgdorferi*sequences**		**No. Hosts*flaB*positive with amplicons homologous to*B. lonestari*sequences**					
		**B31**	**OK HS-2**	**IP2**	**Scc26**	**MO2002-V1**	**TX076**	**NC/MD**	**AA115**
Tick Collections from Canines									
*Amblyomma* species	6/8/62	0	0	0	0	0	1 N, 1 M	1 N, 1 M, 2A	0
*Amblyomma americanum*	105/151/634	0	0	0	2 N, 4 M, 10 F	3 N, 3 M, 9 F	3 N, 2 M, 5 F	15 N, 31 M, 13 F	1 N
*Amblyomma maculatum*	24/26/115	0	0	0	1 N, 1 M	0	8 M, 1 F	12 M, 1 F	0
*Dermacentor variabilis*	17/18/178	0	0	0	2 M, 2 F	1 M, 3 F	3 M, 1 F	3 F	0
*Ixodes scapularis*	28/43/121	0	0	0	1 F	1 M, 1 F	1 M, 4 F	2 N, 11 M, 6 F, 1A	0
*Rhipicephalus sanguineus*	54/78/317	0	0	0	6 N, 8 M, 6 F, 2A	3 N, 2 F	1 M	8 N, 5 M, 3 F, 5A	2 N, 1 F
Total Ticks From Canines	234/324/1427	0	0	0	9 N, 15 M, 19 F, 2A	6 N, 5 M, 15 F	4 N, 16 M, 11 F	26 N, 60 M, 26 F, 8A	3 N, 1 F
Tick Collections from White-tailed Deer									
*Amblyomma* species	1/5/8	1 N	0	0	0	0	0	0	0
*Amblyomma americanum*	2/10/23	0	0	0	0	1 F	0	1 F	0
*Amblyomma maculatum*	15/24/64	1 M	1 F	0	0	2 M, 5 F	1 F	3 M, 2 F	0
*Dermacentor variabilis*	1/1/6	0	0	0	0	1 F	0	0	0
*Ixodes scapularis*	39/209/571	1 M	4 M, 7 F	0	0	6 M, 10 F	2 M, 1 F	4 M, 4 F	0
*Rhipicephalus sanguineus*	4/10/24	0	0	0	0	1 F	1 F	1 M, 1 F	0
Total Ticks From Deer	62/259/696	1 N, 2 M	4 M, 8 F	0	0	8 M, 18 F	2 M, 3 F	8 M, 8 F	0
Ticks, Canines, and White-tailed Deer									
Canine	10/10/173	0	1	6	0	1	0	2	0
Deer	49/49/250	0	21	1	0	3	0	24	0
Total Ticks	296/583/2123	1 N, 2 M	4 M, 8 F	0	9 N, 15 M, 19 F, 2A	6 N, 13 M, 33 F	4 N, 20 M, 14 F	26 N, 68 M, 34 F, 8A	3 N, 1 F
Total	355/642/2546	3	33	7	45	56	38	162	4

^a^ Genbank identities for *Borrelia* strains include strain B31 (AB035617), strain OK HS-2 (FJ871032), strain *IP2* (AY345236), clone Scc26 (DW100451), isolate MO2001-V1 (AY850063), isolate TX076 (EF689742), strain NC/MD (AF273670), and clone AA115 (AY654945).

^b^ Number of larva (L), nymph (N), adult male (M), adult female (F), and damage adult specimens could not be properly sexed (A).

### Identification of *Borrelia* DNA in canines and their ticks

Of the 173 canines and their 1427 ticks subjected to the *flaB* PCR, 5.8% (n = 10) and 22.7% (n = 324) were positive respectively. All ten positive canine *flaB* amplicons were sequenced and six samples were homologous to *B. burgdorferi IP2* (AY345236), two were homolgous to *B. lonestari* NC/MD (AF273670), one was homologous to *B. lonestari* fragments MO2002-V1 (AY850063), and one was homologous to strain *B. burgdorferi* OK HS-2 (FJ871032) (Table 2). Contingency tests revealed that the number of *flaB* positive and negative ticks were significantly different based on tick species (×^2^ = 31.9; df = 5; *P <* 0.0001) such that *I. scapularis* (35.5%) had the highest *flaB* positive rate. The most frequently encountered tick on canines was *A. americanum* and 23.8% (151/634) were *flaB* PCR positive. Of the 173 canines, 87 canines did not have a positive tick, 27 canines had one positive tick, and 59 canines had more than one positive tick. Of the 59 canines with more than one positive tick, 18 canines had multiple specimens of the same species that were positive, 13 canines had specimens of two separate tick species positive, and two canines had specimens of three separate tick species PCR positive.

In canine collected ticks, the *B. lonestari* strain NC/MD (AF273670) was also the most common amplicon and amplified in nymphs, males, and females. Positive ticks were identified primarily in northwest Arkansas where many of the canines were collected (Figure 2A). Ticks from *B. burgdorferi* positive canines did not generate *B. burgdorferi* sequences. Instead these ticks were either negative for *flaB* or produced *flaB* positive amplicons that were homologous with *B. lonestari*.

### Identification of *Borrelia* DNA in white-tailed deer and their ticks

Of the 250 white-tailed deer and the 696 ticks subjected to the *flaB* PCR, 19.6% (n = 49) and 37.2% (n = 259) were positive for *flaB.* Of the 49 white-tailed deer *flaB* amplicons, 24 amplicons were homologous to *B. lonestari* NC/MD (AF273670), 21 amplicons were homologous to *B. burgdorferi* OK HS-2 (FJ871032), 3 amplicons were homolgous to *B. lonestari* MO2002-V1 (AY850063), and one amplicon was homolgous to *B. burgdorferi* IP2 (AY345236) (Table 2). Contingency tests revealed that the number of *flaB* positive and negative ticks were not significantly different based on tick species (X^2^ = 3.96; df = 5; *P* = 0.556). *flaB* prevalence rates in ticks from white-tailed deer was 62.5% (5/8) in unknown *Amblyomma,* 41.7% (10/24) in *R. sanguineus,* 43.5% (10/23) in *A. americanum*, 37.5% (24/64) in *A. maculatum,* 36.6 % (209/571) in *I. scapularis*, and 16.7% (1/6) in *D. variabilis.* A majority of white-tailed deer did not have a positive tick (n = 91), but 70 white-tailed deer had more than one PCR positive tick of which 24 deer had more than one *flaB* positive tick species and one deer had three different tick species *flaB* positive. Fragments amplified from all five deer-collected tick species were most similar to the *B. lonestari* sequence AY850063. Positive ticks were identified throughout Arkansas (Figure 2B).

## Discussion

Borreliae were identified in field collected ticks, canines, and white-tailed deer throughout the state suggesting the bacteria (as a family) are endemic in Arkansas. Of interest was the diversity of *Borrelia* genotypes identified from the variety of different field collections (Figure 1, Table 2). Five different *B. lonestari* genotypes and three different *B. burgdorferi* genotypes were identified in Arkansas. None of the genotypes appeared to be more common in one tick species or host; however, *B. burgdorferi* genotypes were only identified in ticks collected from deer - the significance of this finding remains to be determined. Specifically, *B. burgdorferi* genotypes were amplified only from twelve *I. scapularis,* two *A. maculatum*, and an *Amblyomma* tick that was too damaged for the species to be determined. Only one *I. scapularis* nymph was homologous with *B. burgdorferi,* consequently there is insufficient data to consider the area endemic for Lyme disease. This damaged *Amblyomma* nymph was collected in Washington County of northwest Arkansas. *B. burgdorferi* transmission by *A. maculatum* needs additional attention and previous studies with *A. americanum* have indicated that *A. americanum* cannot transmit *B. burgdorferi*[1,2]. Due to the low prevalence of *B. burgdorferi* in nymphal ticks, Arkansas should not be considered a Lyme disease endemic area [30]. *B. lonestari* NC/MD was the most common sequence amplified (45.6%) and it is homologous to the sequence previously isolated from a patient’s skin with Lyme-like symptoms [41] suggesting this genotype is the most common *Borrelia* in Arkansas. In 2007, four Arkansas counties reported human cases of Lyme disease (Benton, Carroll, Washington, Crawford, and Saline Co.). Ticks collected from these counties in this study primarily produced amplicons homologous to *B. lonestari*, and a few ticks produced amplicons homologous to *B. burgdorferi.* If these human cases of Lyme disease are ‘real’ then they are most likely either rare, acquired from a different location, or are false positives. Collections of field collected ticks near the site of transmission (e.g., where the tick attached to the human) would answer these questions. Data presented here corroborate with Texas collected ticks identified with *B. lonestari* DNA in *A. americanumA. cajennense, A. maculatum, D. variabilis, I. scapularis*, and *R. sanguineus* and relatively few samples of *B. burgdorferi* DNA (AE000783) [39] and with *B. lonestari* in Arkansas white-tailed deer serum [26].

We documented *Borrelia* in five tick species infesting domesticated canines and white-tailed deer; consequently, Borreliae are likely endemic in Arkansas and in the southeastern U.S. by a more diverse tick repertoire than previously thought. Identifying *Borrelia* in ticks indicates that field collected ticks have fed on infected animals at some point in their life cycle and identifying *Borrelia* in canines and white-tailed deer indicates that infected ticks have transmitted *Borrelia* to these hosts. We observed a trend for ticks collected from canines to be positive with *B. lonestari* whereas ticks from deer could be positive with either *B. lonestari* or *B. burgdorferi.* This may be related to the species of tick collected on each host and the sampling periods, as canines were primarily infested with *A. americanum* and collected year round whereas white-tailed deer were primarily infested with *I. scapularis* and collected in the fall. Also, ticks feeding on white-tailed deer may actually be feeding on hosts that are incompetent reservoirs of Lyme disease [42]. Additional evidence is required to assert the potential for each role in transmission. Past research has indicated that several tick species are not active vectors of *B. burgdorferi* (e.g., transmission through salivary glands) [18,20], but additional research should isolate Borrelias from the study area and confirm these tick species are not passive vectors (e.g., transmission studies in a suitable animal model). Additionally, often ticks are misidentified and physicians do not think “beyond Lyme disease” to diagnose the patient properly [2]. Other tick-borne diseases transmitted in Arkansas include rickettsia spotted fevers [32,43] and Ehrlichiosis [13,39], perhaps the cases of Lyme disease reported in 2007 were actually misdiagnosed.

Monitoring tick populations on canines could provide reliable early detection of tick-borne disease outbreaks [44]. Monitoring field populations of ticks for *B. burgdorferi* and other tick-borne diseases is essential to minimize transmission and maximize management. Recent landscape changes in Arkansas are expanding urban environments into deer habitats [45,46]. Previously, this pattern of urban development into previous deer habitats in Lyme Connecticut led to the increased diagnoses of Lyme disease in the northeastern United States [47]; similar reports have indicated *B. burgdorferi* emerged independently in the Midwest and eastern United States [48-50].

Additional research into the involvement of *Borrelia* spp. in the southeastern zoonotic cycle (e.g., abundance of tick/host infection, reservoir maintenance, vector competency) should be conducted in order to determine the life cycle and etiology of the various Borreliae detected in this survey and what roles, if any, they may play relative to subsequent borreliosis observed in humans. Future work should also evaluate the interaction of *A. americanum* carrying *B. lonestari, A. maculatum* carrying *B. burgdorferi*, and *I. scapularis* carrying *B. burgdorferi* and what effect, if any, this might have on the pathogenicity of the Borreliae, as both tick species have been identified simultaneously infesting the same host [27,51]. It is possible that there are multiple interactions occurring between tick species (e.g., species competition for host), the variety of hosts (e.g., Ixodid ticks feed on three different hosts), and the different Borreliae (infesting both the tick and the host), which warrants additional research.

## Conclusions

Data from this study indicate multiple *Borrelia* genotypes are endemic to Arkansas because *flaB* was amplified from ticks, canines, and white-tailed deer, but the exact role each genotype plays in transmission and epidemiology remains undetermined. There were significantly more amplicons of *B. lonestari* than *B. burgdorferi* present suggesting that the majority of tick-borne diseases in Arkansas are not *B. burgdorferi*.

## Abbreviations

flaB, flagellin gene; IFA, indirect fluorescence antibody; IGLUA, Insect Genetics Laboratory at the University of Arkansas; STARI, Southern tick associated rash illness; UNTHSC, University of North Texas Health Science Center in Ft. Worth; VELUA, Veterinary Entomology Laboratory at the University of Arkansas.

## Competing interests

The authors declare we have no competing interests with the work presented in this manuscript.

## Authors’ contributions

RTF, CDS, and ALS conceived the study and designed the experiments. PMB and PCW conducted the work in Texas, and RTF conducted the work in Arkansas. RTF, KLK, PMB, and PCW analyzed the results. RTF and PCW wrote and edited the manuscript. All authors read and approved the final version of the manuscript.
